# A surfactant-free microemulsion composed of isopentyl acetate, *n*-propanol, and water†

**DOI:** 10.1039/c7ra12594a

**Published:** 2018-01-03

**Authors:** Yuan Liu, Jie Xu, Huanhuan Deng, Jiaxin Song, Wanguo Hou

**Affiliations:** State Key Laboratory Base of Eco-chemical Engineering, Qingdao University of Science and Technology 266042 Qingdao P. R. China xujie@qust.edu.cn +86-531-88564750 +86-531-88365460; Key Laboratory for Colloid and Interface Chemistry (Ministry of Education), Shandong University 250100 Jinan P. R. China wghou@sdu.edu.cn

## Abstract

It has been demonstrated that in the absence of traditional surfactants, microemulsions can form from a ternary mixture of oil, water, and an amphi-solvent. These microemulsions are called surfactant-free microemulsions (SFMEs). To date, only a small number of SFME systems have been reported, and the current understanding of SFMEs is very limited. Herein, we report an SFME consisting of isopentyl acetate (IA), *n*-propanol, and water, in which IA (a simple ester compound) and *n*-propanol are used as the oil phase and amphi-solvent, respectively. The microstructures and structural transition of the SFME were investigated by cyclic voltammetry, fluorescence spectroscopy, and UV-visible spectroscopy techniques. Moreover, three kinds of microstructures, namely, oil-in-water (O/W), bicontinuous (BC), and water-in-oil (W/O), have been identified in the SFME, which are directly verified by cryo-TEM observations. A change in the composition of the SFME may lead to a structural transition from O/W through BC to W/O or *vice versa*, which is similar to the case of traditional surfactant-based microemulsions (SBMEs). To the best of our knowledge, this is the first time that the microstructures and structural transition of an SFME obtained using a simple ester compound as the oil phase have been identified.

## Abstract 2

Surfactant-free microemulsions (SFMEs) can form from the mixture of isopentyl acetate (oil phase), *n*-propanol (amphi-solvent), and water. They may show W/O, bicontinuous (BC), and O/W microstructures depending on the composition of the SFMEs.

## Graphical abstract

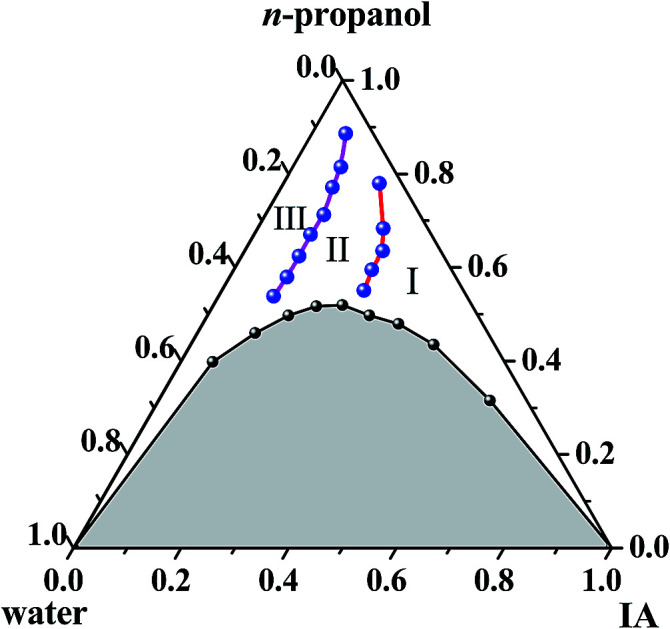

## 1. Introduction

Surfactant-free microemulsions (SFMEs), just as the name implies, are the microemulsions formed in the absence of traditional surfactants.^1,2^ As is well-known, traditional microemulsions are currently defined as thermodynamically stable and optically isotropic transparent dispersions formed by at least two immiscible fluids and a surfactant (or an amphiphilic compound).^3,4^ With reference to the definition of the traditional surfactant-based microemulsions (SBMEs), SFMEs can be defined as thermodynamically stable and optically isotropic transparent dispersions formed by at least two immiscible fluids and an amphi-solvent.^2^ The two immiscible fluids generally consist of a polar and an apolar component, which are traditionally called the water and oil phases, respectively. The amphi-solvent,^2^ an amphiphilic substance but not a traditional surfactant, is a solvent that is completely or at least partially miscible with both the water and oil phases; it cannot form micelles in bulk solutions or ordered films at water–oil interfaces. That is, it has no features of traditional surfactants.

The first SFME was reported by Smith *et al.* in 1977,^4^ and it was found in the ternary system of *n*-hexane, *i*-propanol, and water. Subsequently, the formation of SFMEs was confirmed in some surfactant-free ternary systems.^5–21^ The structures, properties, and formation mechanism of SFMEs have been investigated.^1,2,22–24^ It has been preliminarily demonstrated that the structures and properties of SFMEs are similar to those of traditional SBMEs to some extent.^2^ For instance, similar to SBMEs,^3^ SFMEs can exhibit three structures: oil-in-water (O/W), bicontinuous (BC), and water-in-oil (W/O) structures, and the three kinds of structures can be translated into each other with change in the composition of the system.^7,9–11,13–15,19,21^ In addition, similar to SBMEs,^25^ SFMEs are expected to have extensive potential applications^2^ such as in enzymatic reactions,^26^ chemical reactions,^27–29^ and nanoparticle synthesis.^30–32^ In particular, SFMEs when used as reaction media may avoid some problems, such as complex separation and purification procedures, possible ecotoxicity, and high material cost, caused by the existence of large amounts of surfactants (>10 wt%) in SBMEs.^2^ However, SFMEs have received very less attention as compared to SBMEs.^22^ Over the past few decades, more than 10 000 studies dealing with SBMEs have been reported in the literature,^12^ whereas less than 60 studies dealing with SBMEs have been reported.^2^ In addition, less than 20 SFME systems have been reported to date.^2^ Consequently, the current understanding of SFMEs is still very limited, and many issues about SFMEs need to be studied; one of these issues is to establish whether SFME formation is a general phenomenon. It is desirable to find more SFME systems, which can provide information for understanding the nature of SFMEs.

Herein, we report an SFME consisting of isopentyl acetate (IA), *n*-propanol, and water, in which IA and *n*-propanol are used as the oil phase and amphi-solvent, respectively. The structures and structural transition of the SFME were investigated using cyclic voltammetry, fluorescence spectroscopy, and UV-visible spectroscopy. Similar to the traditional SBMEs, the SFME exhibits W/O, BC, and O/W structures depending on the composition of the ternary system. The three kinds of structures were confirmed by cryogenic transmission electron microscopy (cryo-TEM) observations. The Kunz group^16,20^ has investigated the phase behavior of ester-compound-containing ternary systems including geranyl acetate/ethanol/water^16^ and diethyl adipate/tetrahydrofurfuryl alcohol/deep eutectic solvent (ethylene glycol–choline chloride or urea–choline chloride)^20^ and revealed the formation of SFMEs in these systems by dynamic/static light scattering and small-angle X-ray scattering measurements. However, the microstructures of the SFMEs were not identified. To the best of our knowledge, this is the first study in which the microstructures and structural transition of an SFME obtained using a simple ester compound as the oil phase have been identified. This study provides a better understanding of SFME construction. In addition, the SFME may have some specific applications, such as in material preparation, reaction engineering, and separation, due to its surfactant-free nature.

## 2. Experimental

### 2.1. Chemicals

All the chemicals used in this study were of analytical reagent grade. Isopentyl acetate (IA, purity ≥ 99.5%) and *n*-propanol (purity ≥ 99.8%) were purchased from Sinopharm Chemical Reagent Co., Ltd., China, and their molecular structures are shown in Fig. S1 in the ESI.† Pyrene (GC grade) was purchased from Sigma-Aldrich, USA. Methyl orange (MO) and potassium ferricyanide (K_3_Fe(CN)_6_) were purchased from Tianjin Chemical Reagents Co., China. IA was purified before use by washing with a cold saturated NaCl/NaOH solution (pH = 10–11), drying with 4A molecular sieve, and a final distilling process to remove its free acid impurity. Other chemicals were used as received. Ultrapure water with a resistivity of 18.3 MΩ cm was obtained using an AFZ-1000-U purification system (Chongqing Ever Young Enterprises Development Co. Ltd., China).

### 2.2. Phase diagram construction

The phase diagram of the ternary system IA/*n*-propanol/water was constructed at 25.0 ± 0.2 °C by titration with *n*-propanol for IA/water mixtures. An IA/water mixture with a desired volume ratio (*R*_IA/W_) was prepared in a dry test-tube. An appropriate volume of *n*-propanol was added to the mixture under magnetic stirring. The phase boundary was determined by observing the transition from turbidity to transparency or *vice versa*. Repetition of this experiment for other *R*_IA/W_ values allowed the phase diagram to be established. The entire procedure was repeated three times, and an average value was used. The component content was expressed as the volume fraction in the ternary phase diagram.

### 2.3. Cyclic voltammetry measurements

Cyclic voltammetry measurements were performed using a CHI model 660D electrochemical workstation (Shanghai Chenhua Instrument Factory, China) with a three-electrode cell, which consisted of a glass-carbon working electrode (electrode area 0.07 cm^2^), a AgCl/Ag reference electrode, and a Pt wire counter electrode. The space between adjacent electrodes was 2.0 cm. Before each measurement, the working electrode was polished using a 0.05 mm aluminum oxide slurry and then washed carefully with ultrapure water. The electrode was then ultrasonicated in ultrapure water for approximately 5 min before use. The potential was scanned between −0.2 V and 0.8 V, with a sweep rate range of 20–100 mV s^−1^. All experiments were carried out at 25.0 ± 0.2 °C under a nitrogen atmosphere to avoid oxygen effects. K_3_Fe(CN)_6_ was selected as the electroactive probe, with a fixed concentration of 0.65 g L^−1^.

Based on the peak current data obtained at various sweep rates, the diffusion coefficient of the electroactive probe *D*_p_ (m^2^ s^−1^) in microemulsions can be estimated from the Randles–Sevcik equation:^33^1
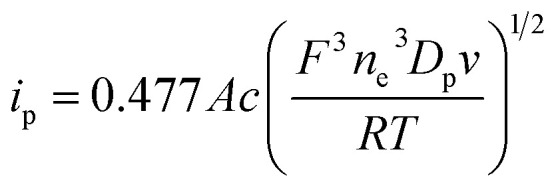
where *i*_p_ is the peak current for a redox-active reversible system, *A* is the area of the working electrode, *c* is the concentration of the electroactive probe, *R* is the gas constant, *T* is the absolute temperature, *F* is the Faraday constant, *n*_e_ is the number of electrons involved in oxidation or reduction, and *v* is the sweep rate. The *D*_p_ of the electroactive probe can be calculated from the slope of an *i*_p_–*v*^1/2^ linear plot.

### 2.4. Fluorescence measurements

Fluorescence emission spectra were obtained using a spectrofluorometer (F-2700, Hitachi, Japan) with a 1 cm path-length quartz cell using pyrene as a fluorescent probe at 25.0 ± 0.2 °C. The pyrene solution in ethanol was placed in a dry test-tube, and ethanol was evaporated by placing the test-tube in a water bath. Appropriate amounts of IA, *n*-propanol, and water were then added to the test-tube, and the pyrene concentration in the mixture was maintained at 4.95 × 10^−5^ mol L^−1^. The excitation and emission slits were set at a 0.5 nm band-pass. The fluorescence emission spectrum of pyrene from 350 to 420 nm was obtained after excitation at 334 nm. The fluorescence emission spectrum of each sample was obtained after allowing sufficient time (∼48 h) for equilibration.

### 2.5. UV-visible adsorption measurements

UV-visible spectra were obtained using a computer-controlled UV-visible spectrometer (TU-1901, Beijing Purkinje General Instrument Co., Ltd., China), with a 1 cm path-length quartz cell using MO as a probe. Appropriate amounts of IA, *n*-propanol, and water containing a desired amount of MO were uniformly mixed in advance, and the resultant sample containing 5.0 mg L^−1^ MO was left for 48 h prior to measurements. The spectrum scanning was carried out at 25.0 ± 0.2 °C with 0.2 nm resolution.

### 2.6. Dynamic light scattering measurements

Dynamic light scattering (DLS) measurements were performed at 25.0 ± 0.2 °C using a Nano ZS90 instrument (Malvern, England) with a 4 mW He–Ne laser (*λ* = 632.8 nm). Before measurements, the samples were filtered through a polycarbonate membrane (0.45 μm pore size) to remove any dust particles. All the scattering photons were obtained at a 90° scattering angle. The scattering data were analyzed using the CONTIN method to obtain the size distribution and average hydrodynamic diameter (*d*_h_) of the test samples. The viscosity and refractive index measurements were carried out using a rotational rheometer (RheolabQC, Anton-Paar, Austria) and an Abbe refractometer (WAY-2S, Shanghai Instrument Physical Optics Instrument Co., Ltd., China), respectively, at 25.0 ± 0.2 °C.

### 2.7. Cryo-TEM observations

Cryogenic transmission electron microscopy (cryo-TEM) observations were performed to determine the structures of microemulsions. The cryo-TEM samples were prepared in a controlled-environment vitrification system (Cryoplunge TM3, USA) at 25 °C and 95% relative humidity. A 4 mL aliquot of the sample was loaded onto a carbon-coated copper grid. The excess solution was then blotted off with filter paper, and a thin film suspended on the mesh holes was produced. After about 5 s, the sample-loaded grid was quickly put in liquid ethane (cooled by liquid nitrogen). The vitrified sample was transferred into a cryogenic specimen holder (Gatan 626) and observed using a JEM-1400 TEM (JEOL, Japan) at approximately −174 °C with an accelerating voltage of 120 kV. The images were obtained using a Gatan multiscan CCD and processed with a Digital micrograph. All samples used for cryo-TEM were filtered through a 0.45 μm filter prior to measurements.

## 3. Results and discussion

### 3.1. Phase behavior of the IA/*n*-propanol/water ternary system

**Fig. 1:**
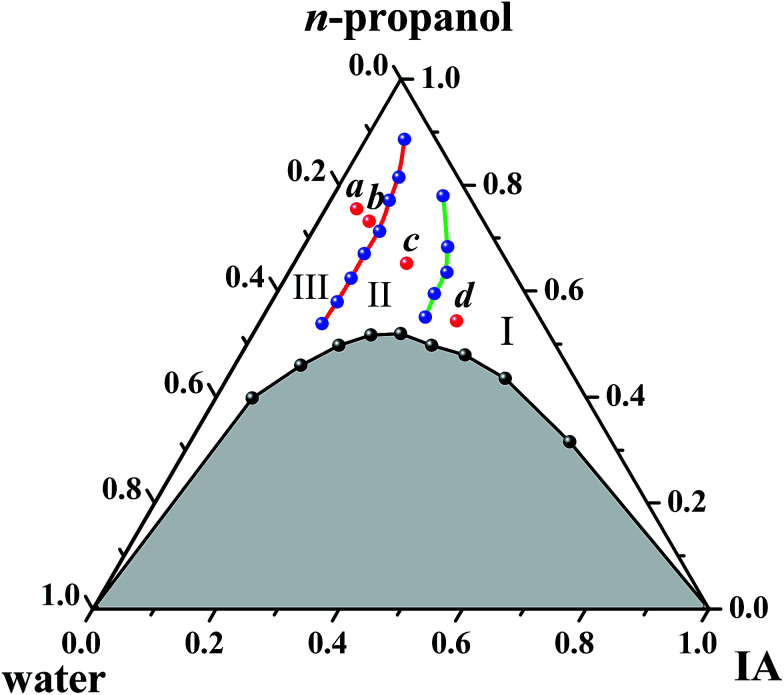
Phase diagram of the IA/*n*-propanol/water system at 25 ± 0.2 °C. I, II, and III represent W/O, BC, and O/W subregions, respectively. Samples *a*, *b*, *c*, and *d* were chosen for cryo-TEM observations.

Fig. 1 shows the ternary phase diagram of the system IA/*n*-propanol/water at 25.0 ± 0.2 °C, in which the component content in the system is expressed as the volume fraction (*f*_i_, where i represents IA, *n*-propanol or water). Moreover, two regions are observed in the diagram: one is a single-phase region (blank region), and the other is a multiphase region (shadow region). In the single-phase region, the ternary systems are optically isotropic and transparent, whereas in the multiphase region, they are turbid under stirring and break quickly into two phases when left to stand. The single-phase region occupies about 55% area in the total phase diagram. This diagram indicates that immiscible IA and water can stably coexist in a homogeneous system with the aid of a certain amount of *n*-propanol. Based on a previous study,^2^ SFMEs most likely form in the single-phase region. In addition, the single-phase channel extending from oil-rich to water-rich regions obtained herein is suitable for studying the structural transition.

### 3.2. Structures and structural transition of microemulsions

#### Cyclic voltammetry

Cyclic voltammetry measurements were performed for the ternary mixtures in the single-phase region using K_3_Fe(CN)_6_ as the electroactive probe to determine the microstructures and structural transition. In the measurements, 20 mM KCl was added in the water phase to improve the conductivity of systems. This low concentration of KCl cannot obviously affect the microstructures of SFMEs.^1,22^ Because only the diffusion-controlled electrochemical charge transfer is suitable for studying the microstructure of microemulsions,^34^ the change in *i*_p_ with *v* has been first determined for two mixtures, in which the volume fraction of IA (*f*_IA_) is 0.05 and the volume ratios of *n*-propanol to water (*R*_P/W_) are 8.0/2.0 and 7.0/3.0. The *i*_p_–*v*^1/2^ plots obtained for the two mixtures are all straight lines passing through the origin (Fig. S2, ESI†), indicating that the electron transport of the electrode reaction in the mixtures is diffusion-controlled.^35,36^ Therefore, K_3_Fe(CN)_6_ is a suitable electroactive probe for studying the microstructures and structural transition of the microemulsions.

**Fig. 2:**
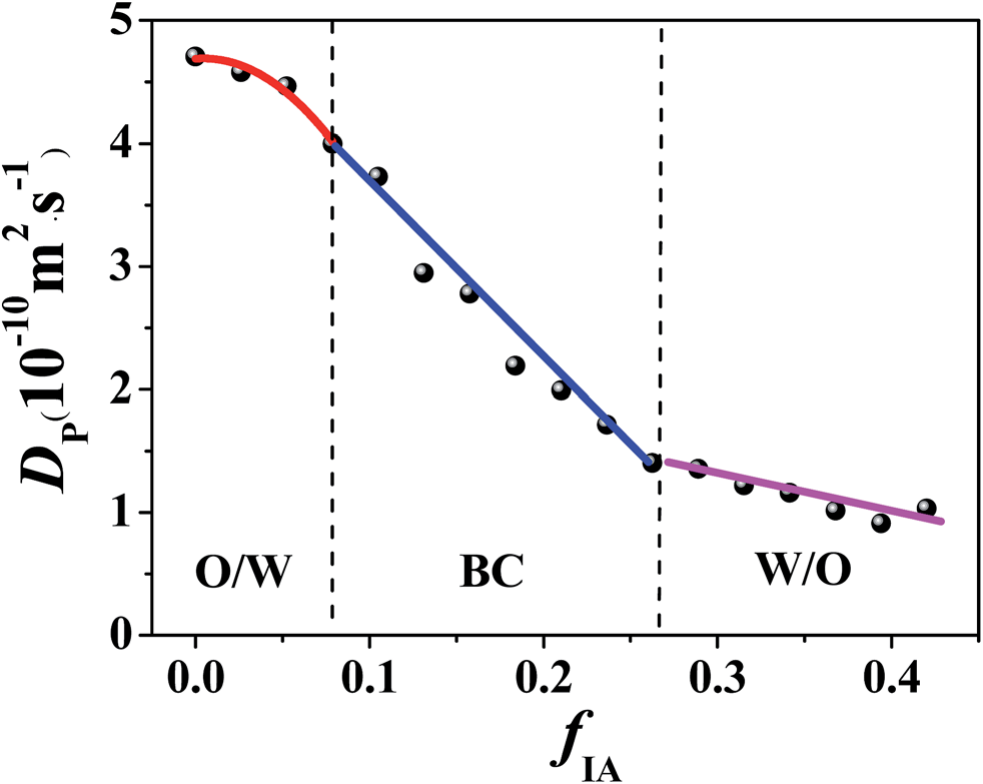
Diffusion coefficient of K_3_Fe(CN)_6_ in the IA/*n*-propanol/water ternary system with *R*_P/W_ = 8.0/2.0 as a function of *f*_IA_. The K_3_Fe(CN)_6_ concentration is 0.65 g L^−1^.

Along various IA dilution lines with different *R*_P/W_ values (Fig. S3, ESI†), the change in *D*_p_ of K_3_Fe(CN)_6_ in the ternary system over the single-phase region was examined. A typical plot of *D*_p_*versus f*_IA_ for *R*_P/W_ = 8.0/2.0 is shown in Fig. 2. The *D*_p_ decreases monotonically with the increasing *f*_IA_ over the entire single-phase region. However, two breakpoints are observed in the *D*_p_–*f*_IA_ plot. The whole curve can therefore be divided into three successive stages: an initial slow decrease, a subsequent quicker decrease, and a final slower decrease, as indicated in Fig. 2. Similar results are obtained for *R*_B/P_ = 9.5/0.5, 9.0/1.0, 8.5/1.5, and 7.5/2.5 (Fig. S4, ESI†). Based on previous studies,^14,15,33,37,38^ the discontinuous change in *D*_p_ can be attributed to the change in the microstructure of the K_3_Fe(CN)_6_-located environment, *i.e.*, a microemulsion forms in the ternary system and its microstructure is changed with a change in *f*_IA_. Generally, when an electroactive probe is completely solubilized in dispersed droplets, a lower *D*_p_ value will be obtained in comparison with that from a continuous phase. This is because the probe located in droplets diffuses with the droplets, and the obtained *D*_p_ value corresponds to the apparent diffusion coefficient of the droplets.^37,38^ In this study, the electroactive probe K_3_Fe(CN)_6_ is expected to preferentially probe the water environment because of its limited solubility in IA. Therefore, the high *D*_p_ value at low *f*_IA_ (*e.g.*, <0.08 for *R*_P/W_ = 8.0/2.0) suggests the formation of an O/W type microemulsion, which corresponds to the diffusion of the probe in the water-rich continuous phase. The low *D*_p_ value at high *f*_IA_ (*e.g.*, *f*_IA_ > 0.27 for *R*_P/W_ = 8.0/2.0) suggests the formation of a W/O type microemulsion, which corresponds to the diffusion of the water-rich droplets. At intermediate *f*_IA_ (*e.g.*, 0.08 < *f*_IA_ < 0.27 for *R*_P/W_ = 8.0/2.0), the change rate in *D*_p_ with *f*_IA_ is different from those at both high and low *f*_IA_; this suggests the formation of a BC microstructure.^14,15,33,35,37,39,40^

The cyclic voltammetry data demonstrate that with the increasing *f*_IA_, a structural transition of the microemulsion occurs, *i.e.*, from O/W through BC to W/O. Notably, only one breakpoint appears for the ternary systems with *R*_P/W_ = 7.0/3.0, 6.5.0/3.5, and 6.0/4.0 (Fig. S4, ESI†), suggesting that the transition from W/O to BC occurs along the IA dilution lines. Furthermore, no obvious breakpoint appears for the ternary system with *R*_P/W_ = 5.5/4.5 (Fig. S4, ESI†); this suggests that no phase transition occurs along the IA dilution line.^14,21^

#### Fluorescence spectroscopy

**Fig. 3:**
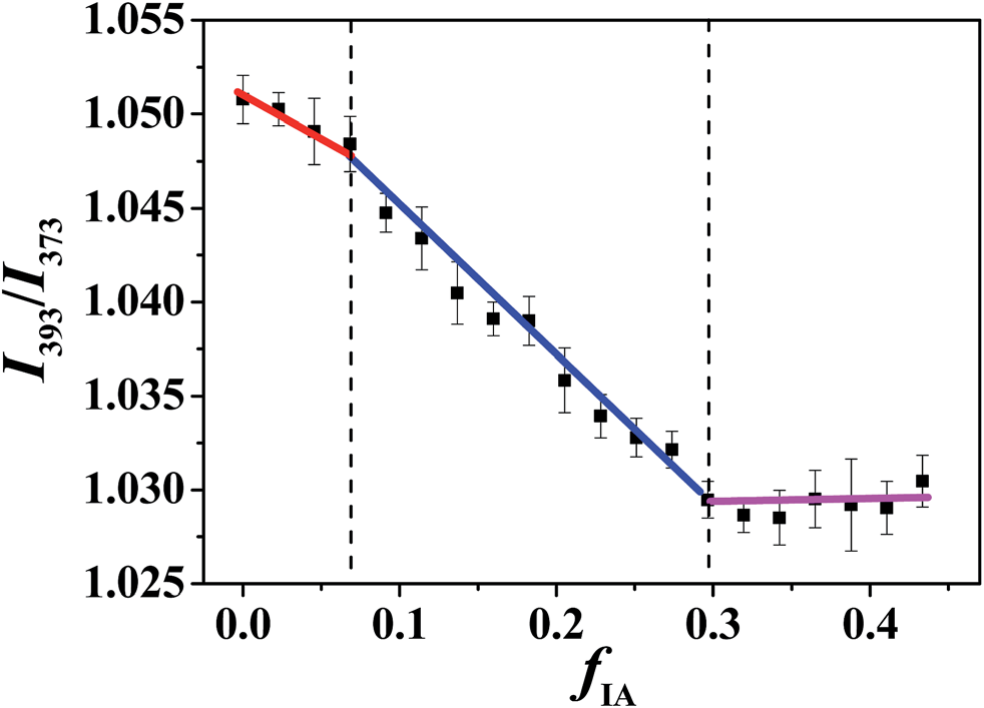
Intensity ratio *I*_393_/*I*_373_ of pyrene in microemulsions at *R*_P/W_ = 8.0/2.0 as a function of *f*_IA_. The pyrene concentration is 4.95 × 10^−5^ mol L^−1^.

Hydrophobic pyrene is a widely used fluorescence probe, and its fluorescence emission spectrum shows five vibronic bands at 373, 378, 383, 388, and 393 nm. The intensity ratio of the fifth peak to the first peak, *I*_393_/*I*_373_, is very sensitive to the polarity of pyrene-located microenvironments. A high *I*_393_/*I*_373_ value represents a low polarity of the microenvironment.^41,42^ In our previous study,^15,21^ we have found that a discontinuous change in *I*_393_/*I*_373_ with *f*_w_ or *f*_o_ may occur as the microstructure of microemulsion changes. Therefore, fluorescence spectroscopy of pyrene can be used to identify the structural transition of microemulsions. Along various IA dilution lines with different *R*_P/W_ values (9.0/1.0, 8.0/2.0, and 7.0/3.0), the change in *I*_393_/*I*_373_ of pyrene in the microemulsion was determined. Fig. 3 shows a typical curve of *I*_393_/*I*_373_*versus f*_IA_ for *R*_P/W_ = 8.0/2.0. The *I*_393_/*I*_373_ value decreases monotonically with the increasing *f*_IA_, and two breakpoints are clearly observed. Similar results are obtained for *R*_P/W_ = 9.0/1.0 (Fig. S5C, ESI†).

The *I*_393_/*I*_373_ values of pyrene in bulk water, IA, and *n*-propanol were determined to be 0.824, 1.035, and 1.075, respectively, indicating that the order of the polarity for the three components is water > IA > *n*-propanol. In addition, hydrophobic pyrene is expected to preferentially probe the IA microenvironment because of its limited solubility in water. Notably, the IA and water phases in the SFMEs studied herein actually consist of IA/*n*-propanol and water*/n*-propanol solutions, respectively, owing to the fact that *n*-propanol is miscible with both IA and water. With the increasing *f*_IA_, the *n*-propanol content in the IA/*n*-propanol phase decreases; this may lead to a decrease in *I*_393_/*I*_373_ owing to the *I*_393_/*I*_373_ value of *n*-propanol being higher than that of IA. In addition, the *f*_IA_ values corresponding to the breakpoints are very close to those observed in cyclic voltammetry measurements (Fig. S5, ESI†). Therefore, the discontinuous change in *I*_393_/*I*_373_ observed herein can be attributed to the structural transition of the microemulsion with changing *f*_IA_. That is, the three changing stages in *I*_393_/*I*_373_ observed from low to high *f*_IA_ correspond to W/O, BC, and O/W microstructures. Notably, only one breakpoint appears for the ternary system with *R*_P/W_ = 7.0/3.0 (Fig. S6B, ESI†), suggesting that only a transition from W/O to BC occurs along the IA dilution line, which is similar to the cyclic voltammetry result.

#### UV-visible spectroscopy

**Fig. 4:**
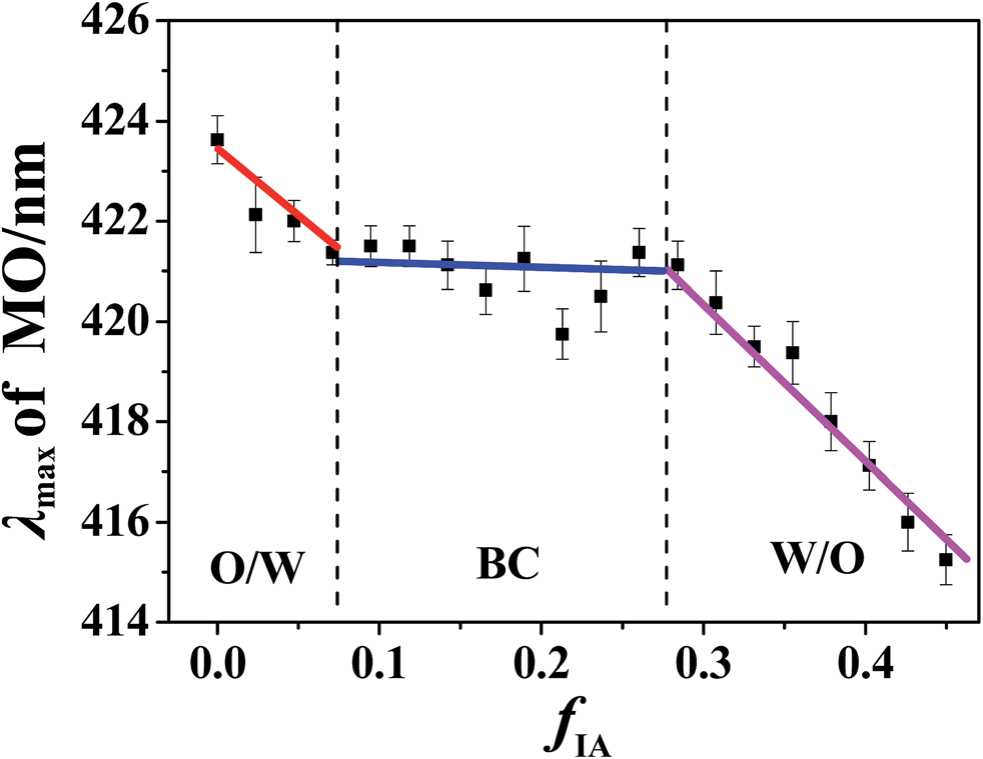
MO *λ*_max_ as a function of *f*_IA_ in microemulsions at *R*_P/W_ = 8.0/2.0. The MO concentration in microemulsions is 5 mg L^−1^.

MO is a widely used dye probe, and its visible absorption maximum (*λ*_max_) is sensitive to its local microenvironment.^33,43^ A high polarity of MO-located microenvironments may result in a high *λ*_max_ value. In our previous study,^15,21^ we have found that a discontinuous change in *λ*_max_ with *f*_w_ or *f*_o_ may occur as the microstructure of microemulsion changes. Therefore, UV-visible spectroscopy of MO can be used to identify the structural transition of microemulsions. Along various IA dilution lines with the given *R*_P/W_ values (9.0/1.0, 8.0/2.0, and 7.0/3.0), the change in *λ*_max_ of MO in the microemulsions was determined. Fig. 4 shows a typical curve of *λ*_max_*versus f*_IA_ for *R*_P/W_ = 8.0/2.0. With the increasing *f*_IA_, the *λ*_max_ value initially decreases, then remains almost unchanged, and finally decreases again. Moreover, two breakpoints appear in the whole *λ*_max_–*f*_IA_ plot. A similar result is obtained for *R*_P/W_ = 9.0/1.0 (Fig. S5E, ESI†). MO is expected to preferentially probe the water microenvironment due to its good water-solubility. In addition, MO prefers to locate at the water/IA interfacial phase,^15,21^ thereby being also sensitive to the IA content. The *λ*_max_ value of MO in bulk water (464.2 nm) is obviously higher than that in bulk IA (416.4 nm). Therefore, the increase of *f*_IA_ leads to a decrease in *λ*_max_ of MO. The almost unchanged *λ*_max_ at intermediate *f*_IA_ suggests that the corresponding microstructure is different from those formed at low and high *f*_IA_. Furthermore, the *f*_IA_ values corresponding to the breakpoints are very close to those observed in cyclic voltammetry and fluorescence spectroscopy measurements (Fig. S5, ESI†). Therefore, it can be concluded that the three changing stages in *λ*_max_ observed from low to high *f*_IA_ correspond to the formation of W/O, BC, and O/W microstructures. Similar to the cyclic voltammetry and fluorescence results, only one breakpoint is observed for *R*_P/W_ = 7.0/3.0 (Fig. S6C, ESI†).

#### Subregions of single-phase microemulsion region

The abovementioned results of cyclic voltammetry and fluorescence and UV-visible spectroscopies demonstrate that the IA/*n*-propanol/water ternary system can form microemulsions. With the increasing *f*_IA_, the microstructures of the microemulsions can translate from W/O through BC to O/W. The boundaries for the three microstructures obtained by the three techniques are consistent with each other (Fig. S5 and S6, ESI†). The three subregions corresponding to the W/O, BC, and O/W microemulsions, respectively, are marked in Fig. 1. The uncertainty in locating the boundaries of different subregions is estimated to be less than 10%. The microstructures and structural transition observed herein are similar to those reported for other SFMEs^2,7,9,13–15,19,21^ and SBMEs.^33,35,38–40^

### 3.3. Cryo-TEM and DLS studies

**Fig. 5:**
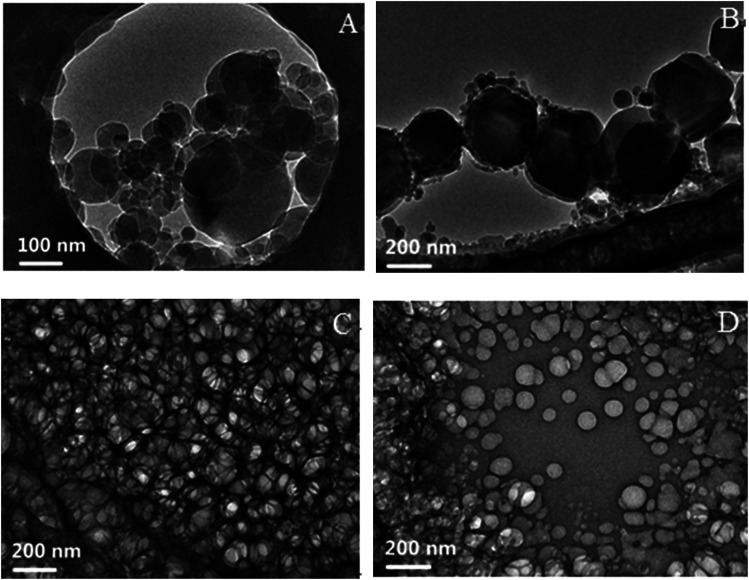
Cryo-TEM images of the samples (A) *a*, (B) *b*, (C) *c*, and (D) *d*. The samples *a* and *b* fall in the O/W subregion, and the samples *c* and *d* fall in the BC and W/O subregions, respectively, as marked in Fig. 1.

Along the IA dilution line with *R*_P/W_ = 8.0/2.0, four samples with *f*_IA_ = 0.050, 0.083, 0.183, and 0.318 (denoted as *a*, *b*, *c*, and *d*, respectively) were chosen for cryo-TEM observations. The samples *a* and *b* fall in the O/W subregion, and the samples *c* and *d* fall in the BC and W/O subregions, respectively, as marked in Fig. 1. The cryo-TEM images of the four samples are shown in Fig. 5 (and Fig. S7, ESI†). Spherical droplets are observed for the samples *a*, *b*, and *d*, demonstrating the existence of discrete droplets in the systems. The average diameters of droplets for the samples *a*, *b*, and *d* are measured to be ∼80, 200, and 120 nm, respectively. For the sample *c*, a network structure (or a sponge-like structure) formed by the interconnection of droplets is observed, corresponding to both IA and water being continuous phases in the microstructure. The cryo-TEM results directly verify the formation of W/O, BC, and O/W microemulsions.

**Fig. 6:**
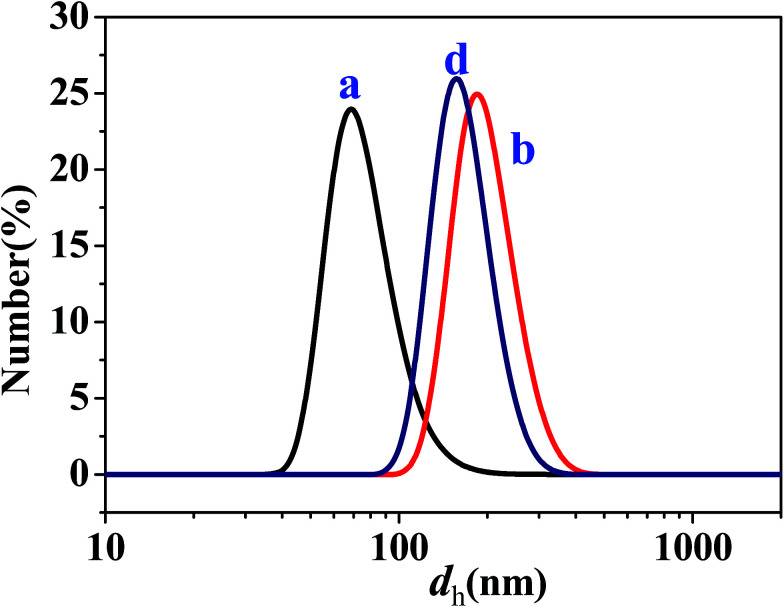
Size distributions of dispersed droplets for the samples *a*, *b*, and *d* as marked in Fig. 1.

To further determine the sizes of the dispersed droplets in the W/O and O/W SFMEs, DLS measurements were carried out for the samples *a* and *b* in the O/W subregion and the sample *d* in the W/O subregion (as marked in Fig. 1), as shown in Fig. 6. The *d*_h_ values of the samples *a*, *b*, and *d* are 77, 198, and 167 nm, respectively, close to their sizes observed by cryo-TEM.

### 3.4. Stability of microemulsions

The stability of the microemulsions was examined by the following processes: (1) heat-storage at 80 °C for one week; (2) cold-storage at −10 °C for one week; (3) freeze (−10 °C)-thaw (40 °C) cycle 3 times; and (4) centrifugation under 100*g* for 15 min. No changes in the appearance of the microemulsion systems are found after different treatments, which is consistent with the thermodynamic stability characteristics of microemulsions.

Notably, the mechanism of the SFME formation is still unclear. The decrease in the isopentyl acetate (IA)/water interfacial tension caused by the addition of *n*-propanol may play a key role in the formation of the SFME. Most likely, *n*-propanol is preferentially located in the interphase between IA and water, as suggested in the previous reports.^5,20^ Many essential questions concerning the nature of SFMEs remain to be addressed.

## 4. Conclusion

The phase diagram of the ternary mixture of IA (oil phase), *n*-propanol (amphi-solvent), and water shows a single-phase and a multiphase region. Microemulsions, *i.e.*, SFMEs, form in the single-phase region. The SFMEs may exhibit O/W, BC, and W/O microstructures, which are directly confirmed by cryo-TEM observations. A change in the composition of the SFMEs may lead to a structural transition from O/W through BC to W/O or *vice versa*, which is similar to the case of traditional SBMEs. This study provides evidence for the speculation that SFME formation may be a general phenomenon. These SFMEs may have specific applications in material preparation, reaction engineering, and separation because of their surfactant-free nature.

## Conflicts of interest

The authors declare no competing financial interest.

## Supplementary Material

RA-008-C7RA12594A-s001
