# Supplementary material for: A surfactant-free microemulsion composed of isopentyl acetate, n-propanol, and water
Source: RSC Adv. 2018 Jan 3;8(3):1371–7. doi: 10.1039/c7ra12594a (PMC9077043; doi:10.1039/c7ra12594a)
Supplement: RA-008-C7RA12594A-s001 [file RA-008-C7RA12594A-s001.pdf]

Electronic Supplementary Material (ESI) for RSC Advances.
This journal is © The Royal Society of Chemistry 2017

Supporting Information

Surfactant-Free Microemulsion Composed of Isopentyl Acetate, *n*-Propanol, and Water

Yuan Liu [a], Jie Xu * [a], Huanhuan Deng [a], Jiaxin Song [a], Wanguo Hou * [b]

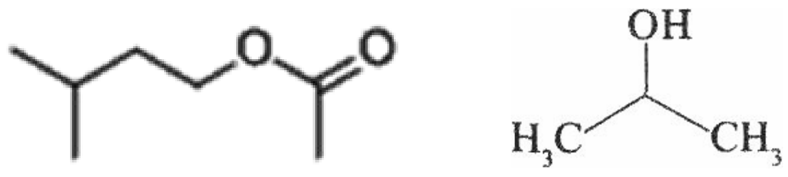

(a) **(b)**

Fig. S1. Molecular structures of (a) isopentyl acetate and (b) *n*-propanol.

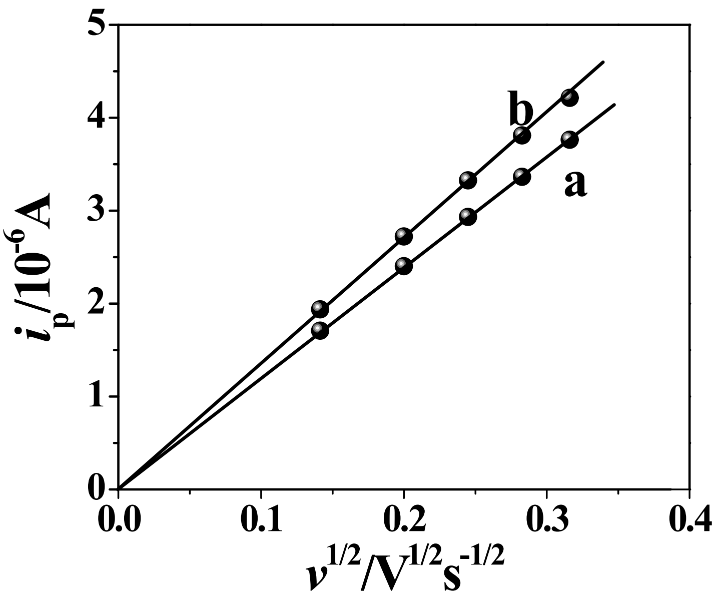

Fig. S2. Scan-rate dependence of anodic peak currents in microemulsions at f_{IA}=0.050 with (a) $R_{P/W}$ = 8.0/2.0 and (b) $R_{P/W}$ = 7.0/3.0.

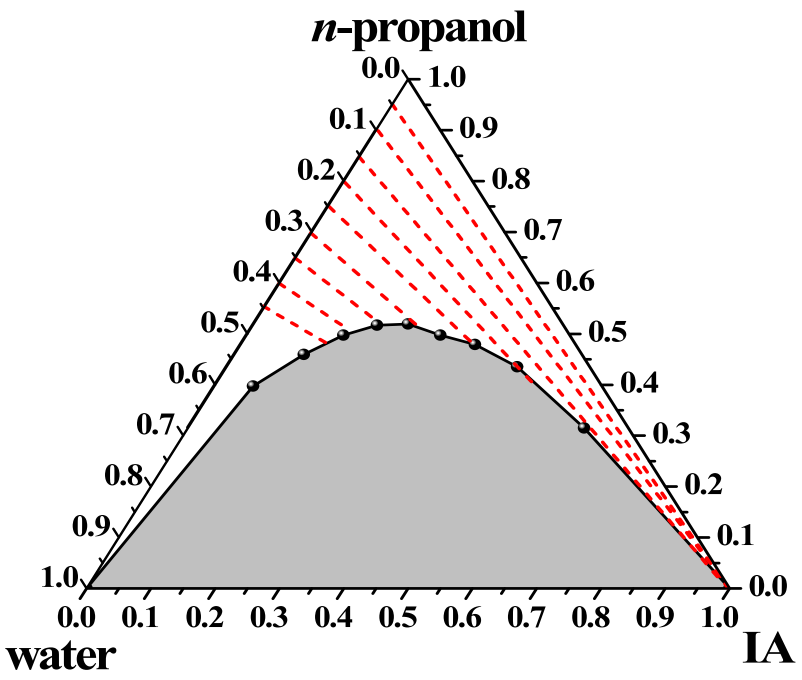

Fig. S3. IA dilution lines with different $R_{P/W}$ values for cyclic voltammetry, fluorescence spectroscopy, and UV-visible spectroscopy mesurements

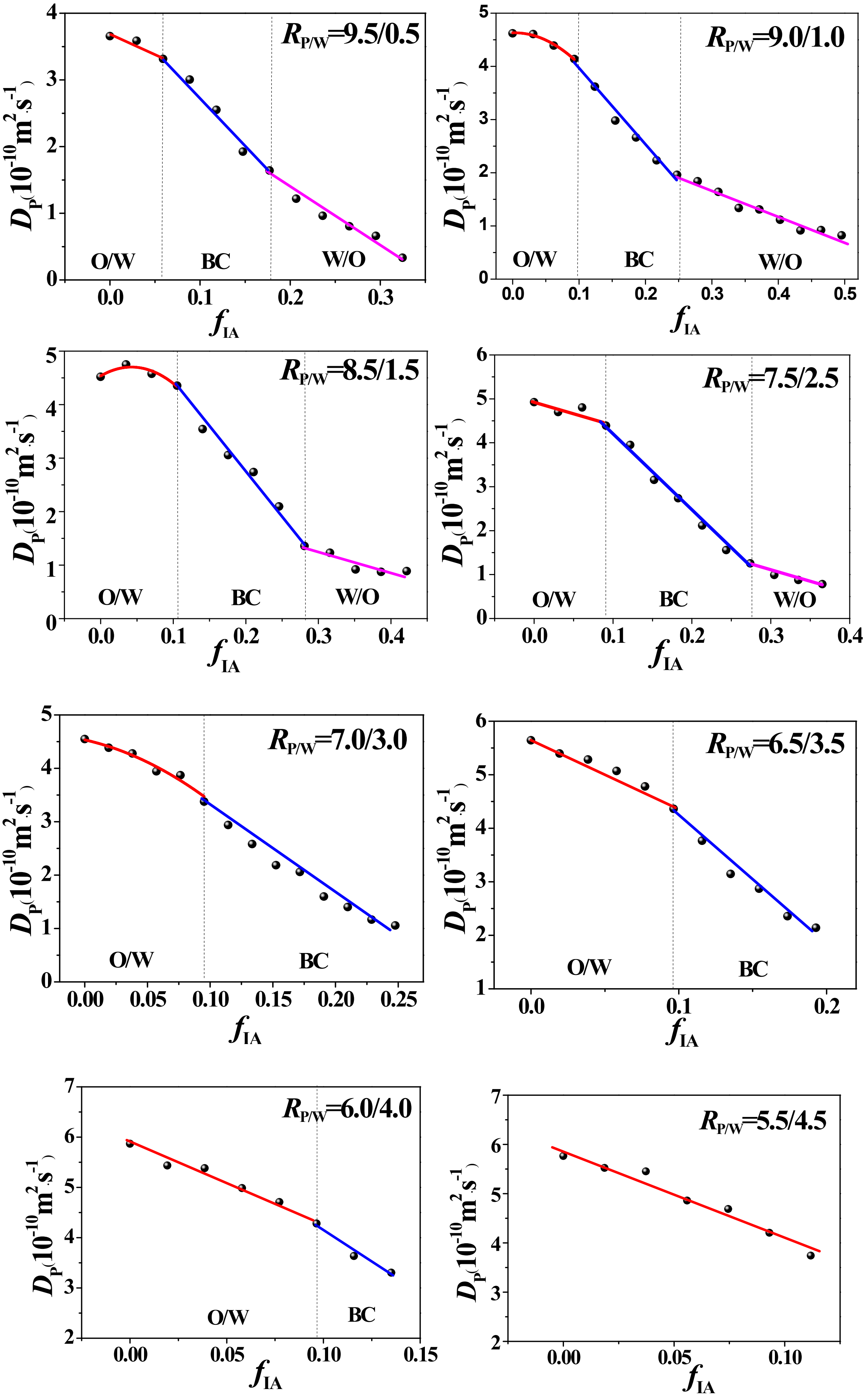

Fig. S4. Diffusion coefficient (D_p) of $K_3Fe(CN)_6$, in microemulsions as a function of f_{IA} at various $R_{P/W}$ .The concentration of $K_3Fe(CN)_6$ was 0.65 g·L^{-1}.

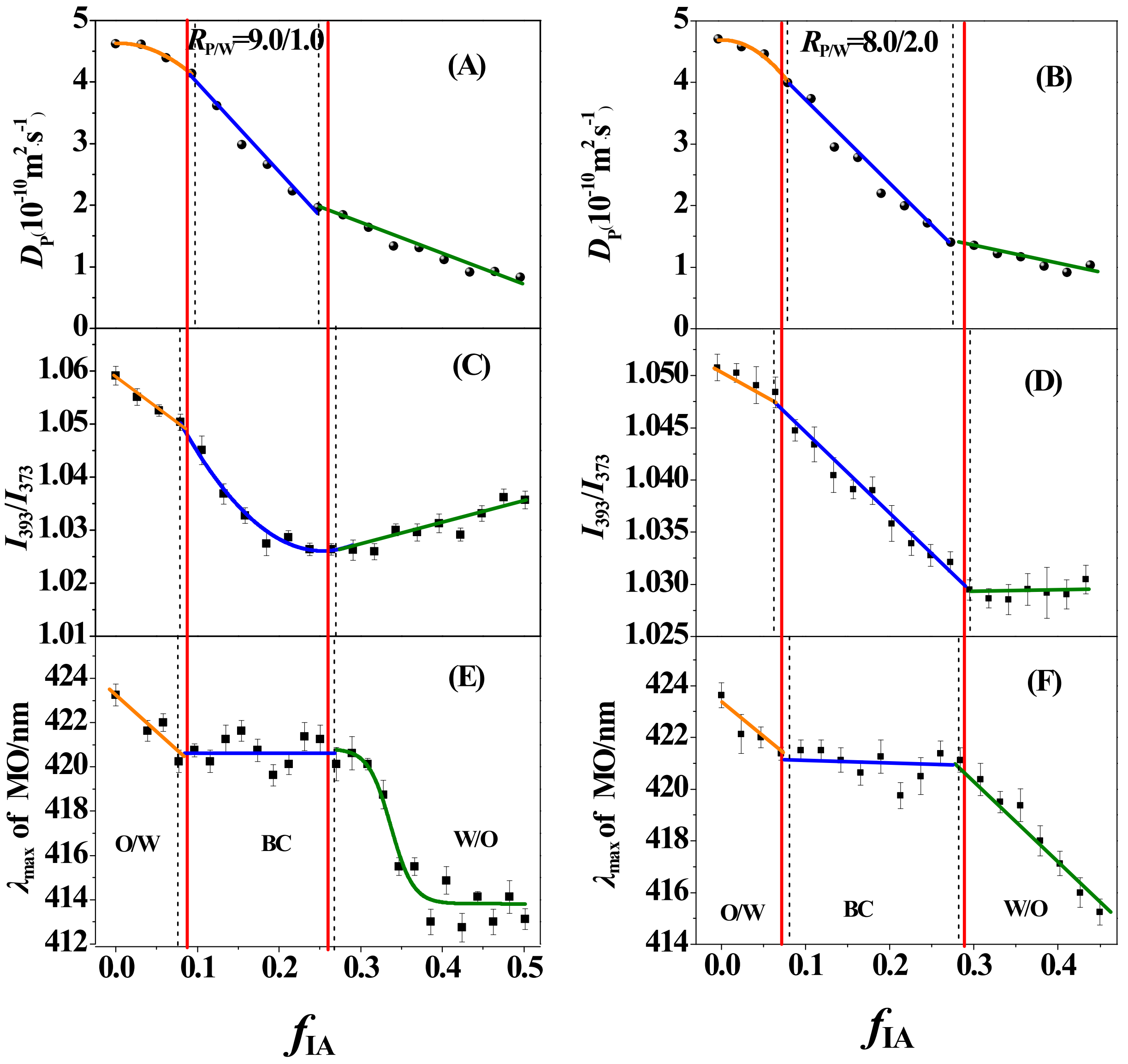

Fig. S5. (A, B) D_p of $K_3Fe(CN)_6$, (C, D) I_{393}/I_{373} of pyrene, and (E, F) λ_{max} of MO in microemulsions at (A, C, E) $R_{P/W}$ = 9/1 and (B, D, F) $R_{P/W}$ = 8.0/2.0 as a function of f_{IA}.

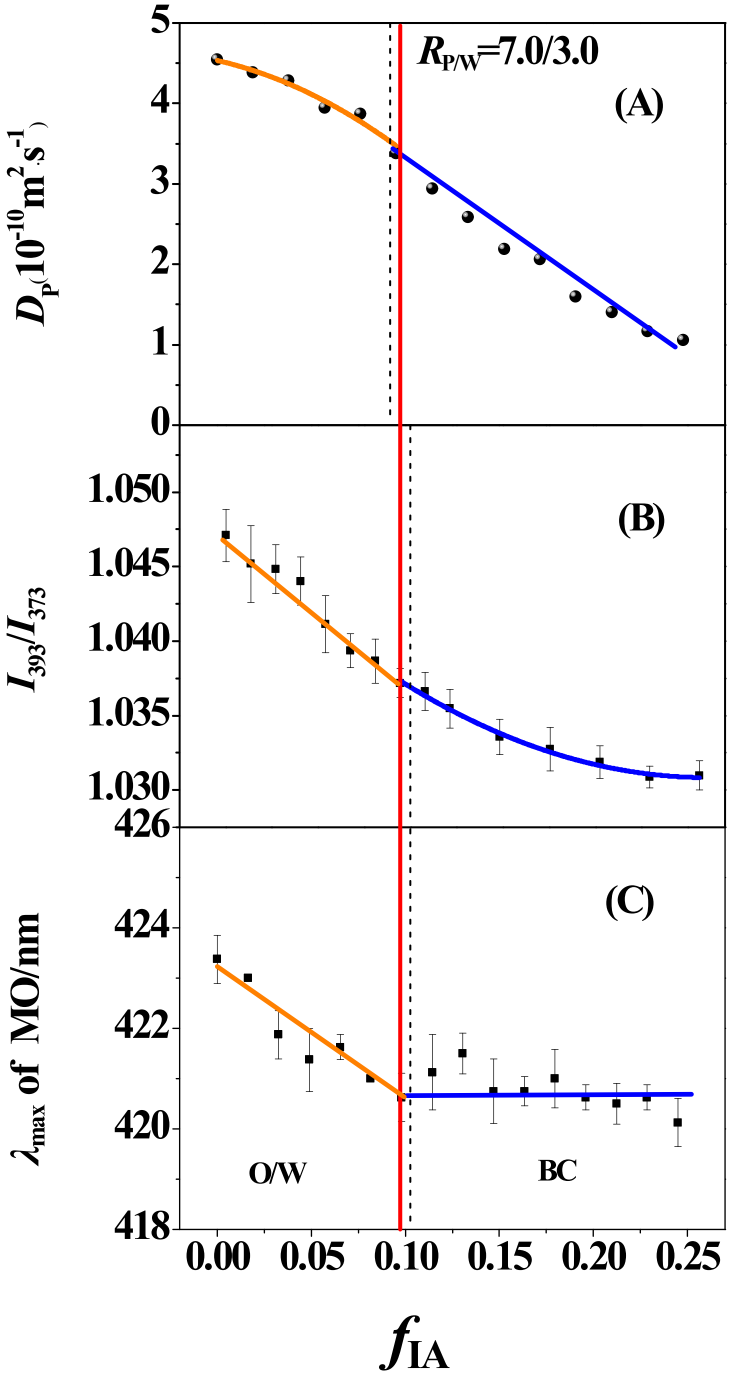

Fig. S6. (A) D_p of $K_3Fe(CN)_6$, (B) I_{393}/I_{373} of pyrene, and (C) λ_{max} of MO in microemulsions at $R_{P/W}$ = 7.0/3.0 as a function of f_{IA}.

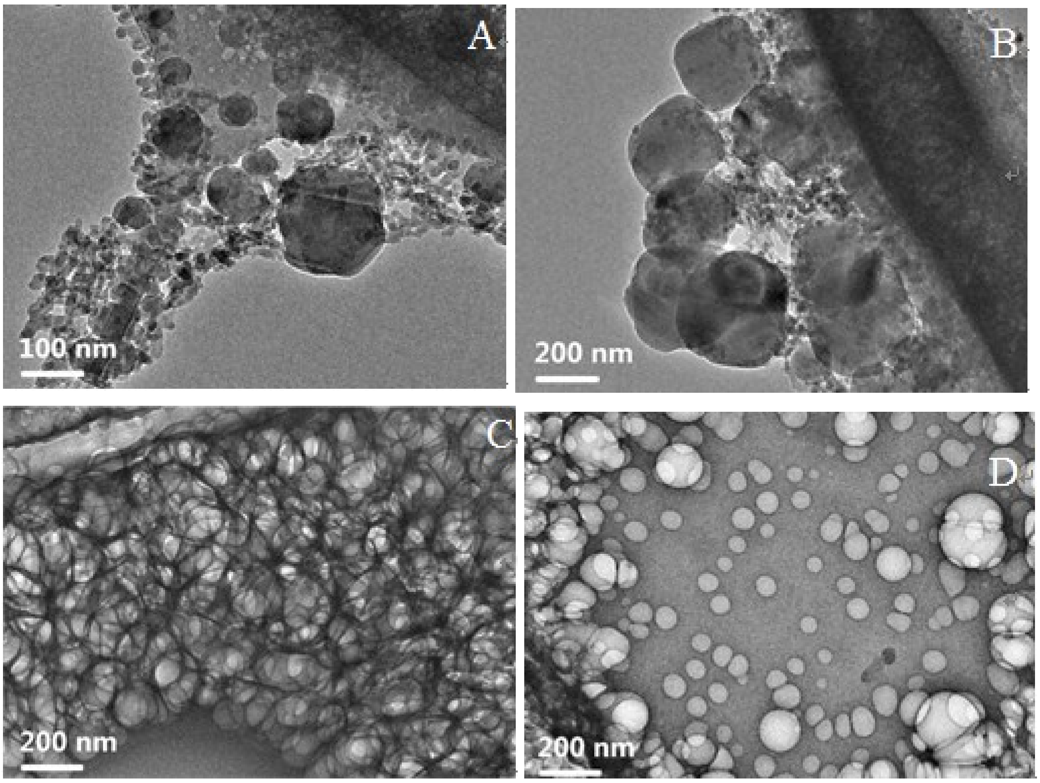

Fig. S7. Cryo-TEM images of samples (A) *a*, (B) *b*, (C) *c*, and (D) *d*. The samples *a* and *b* fall in the O/W subregion, and the samples *c* and *d* fall in the BC and W/O subregions, respectively, as marked in Fig. 1.
